# First isolation of a methanotrophic *Mycobacterium* reveals ammonia- and pH-tolerant methane oxidation

**DOI:** 10.1128/aem.00796-25

**Published:** 2025-07-31

**Authors:** Hiromi Kambara, Taito Kawamoto, Shuji Matsushita, Tomonori Kindaichi, Noriatsu Ozaki, Yoshiteru Aoi, Yoshihiro Takaki, Hiroyuki Imachi, Masaru Konishi Nobu, Miyuki Ogawara, Akiyoshi Ohashi

**Affiliations:** 1 Institute for Extra-cutting-edge Science and Technology Avant-garde Research (X-star), Japan Agency for Marine-Earth Science and Technology (JAMSTEC), Yokosuka, Kanagawa, Japan; 2 Department of Civil and Environmental Engineering, Graduate School of Advanced Science and Engineering, Hiroshima University 12803, Higashi-Hiroshima, Japan; 3 Agricultural Technology Research Center, Hiroshima Prefectural Technology Research Institute 175722, Higashi-Hiroshima, Japan; 4 Program of Biotechnology, Graduate School of Integrated Sciences for Life, Hiroshima University 592294 , Higashi-Hiroshima, Japan; Georgia Institute of Technology, Atlanta, Georgia, USA

**Keywords:** methanotroph, *Mycobacterium*, ammonia, enrichment, isolation

## Abstract

Methanotrophs facilitate a key step in the global carbon cycle—oxidation of natural and anthropogenic methane—and comprehension of their physiology is critical in unraveling the ecology revolving around microbial methane sinks. Recent literature on aerobic methanotroph physiology focuses on two phyla (*Pseudomonadota* and *Verrucomicrobiota*) containing methane-oxidizing isolates; however, such studies no longer discuss a group of gram-positive methanotrophs proposed to belong to *Actinomycetota* (formerly Actinobacteria). Despite their identification four decades ago, cultures have been lost, leaving their biology and sequenced-based taxonomy enigmatic. Here, we report isolation and characterization of a gram-positive methanotroph, strain MM-1, belonging to *Mycobacterium* of the phylum *Actinomycetota*. This marks *Actinomycetota* as the third phylum (and the only phylum of the kingdom *Bacillati*) containing isolated aerobic methanotrophs. Unlike conventional methanotrophs, MM-1 demonstrated a remarkably broad pH tolerance and high resistance to a ubiquitous competitive inhibitor of methanotrophy, ammonia. MM-1 possesses a canonical soluble methane monooxygenase and lacks known genes for the degradation of hydroxylamine (a toxic byproduct of ammonia oxidation), suggesting a novel NH_3_ tolerance mechanism. The unique tolerance to ammonia would allow *Actinomycetota* methanotrophs to serve as methane sinks in various settings where conventional methanotrophs would be challenged. A 16S rRNA gene-based survey showed that sequences similar to MM-1 were detected in various environments, including drinking water systems, where the ammonia-to-methane ratio is high, posing challenges for conventional methanotrophs. Through the first isolation of *Actinomycetota* methanotrophs, our study broadens the recognized physiological range of methanotrophs and suggests the existence of overlooked ecological niches tied to methane oxidation.

**IMPORTANCE:**

Methane is a significant contributor to climate change (27 times more potent as a greenhouse gas than carbon dioxide), and the largest biological sink is methane-oxidizing bacteria: methanotrophs. Although such organisms are thought to be phylogenetically diverse, so far, physiological characterization has been limited to a few clades for which isolates have been captured. Here we successfully obtain the first aerobic methanotrophic isolate outside the phyla *Pseudomonadota* and *Verrucomicrobiota*, from the genus *Mycobacterium* of the phylum *Actinomycetota*. This isolate exhibited remarkable tolerance to a universal inhibitor of methanotrophy, ammonia, well above concentrations where conventional methanotrophy stops. This expands our understanding of the ecological significance of *Mycobacterium*, which is generally associated with the medical field for pathogenicity. Our discovery shows that methanotrophy may contribute to mitigating methane emissions in a wider range of environmental conditions than previously thought, providing critical insight for comprehensive evaluation of methanotrophy’s contributions to the global methane cycle.

## INTRODUCTION

Methane is a potent greenhouse gas that significantly contributes to climate change, with a global warming potential approximately 27 times greater than that of CO_2_ over a 100-year timescale (1). Atmospheric methane concentrations have been rising rapidly (2). Methane emissions arise from both natural and anthropogenic sources (3). Natural sources include wetlands, freshwater systems, termites, geological seepage, and the ocean. Anthropogenic sources stem from livestock, landfills, rice cultivation, fossil fuels, and biomass burning. The major sinks of atmospheric methane include oxidation by hydroxyl radicals (4) and microbial oxidation (5). One group of bacteria that plays a crucial role in mitigating methane emissions is methanotrophs: methane-oxidizing bacteria that utilize methane as their sole carbon and energy source (6).

Methanotrophs were initially described as aerobic, mesophilic, neutrophilic, and associated with the phylum *Pseudomonadota*, within the classes *Gammaproteobacteria* (type I) and *Alphaproteobacteria* (type II) (5), but efforts in cultivation have shown methanotrophs to be more phylogenetically and physiologically diverse and thereby expanding our understanding of the biogeochemical contributions of methanotrophy. Newly identified *Verrucomicrobia* could perform methanotrophy in low-pH high-temperature habitats (i.e., acidic geothermal environments) (7) and species of *Candidatus* (*Ca.*) Methylomirabilota (NC10) were shown to perform aerobic methanotrophy in anoxic habitats by producing their own oxygen (8). Moreover, recent metagenomic studies have found potential evidence for methanotrophy in a wider phylogenetic range of bacteria (e.g., Bay et al. [9]), suggesting other unknown physiological diversity exists.

Until several decades ago, there were reports of gram-positive methanotrophs inferred to belong to *Mycobacterium* and *Actinomyces* of the phylum *Actinomycetota* (formerly Actinobacteria) based on morphology (10–12). However, the taxonomic identity of these organisms remains uncertain due to the lack of molecular information and deposited/preserved cultures. As such, most current literature on aerobic methanotrophic bacteria strictly discusses isolates belonging to *Pseudomonadota* and *Verrucomicrobiota*. Recently, discussion of methanotrophic *Actinomycetota* was reignited by a study reporting a mixed methane-oxidizing culture predominated by *Mycobacterium* and compelling isotopic evidence of methanotrophy by this population (13). However, the physiological properties of methanotrophic *Mycobacterium* and how these may distinguish them from conventional methanotrophs remain unclear due to a lack of isolates. Given the ubiquity and ecological importance of *Actinomycetota*, we may be overlooking a major component of methane cycling and associated ecological processes.

In this study, we successfully obtained a methanotrophic *Mycobacterium* strain of the phylum *Actinomycetota*, designated MM-1, through enrichment in a continuous-flow reactor and subsequent colony isolation. While most methanotrophs are thought to be metabolically sensitive to a ubiquitous inhibitor, ammonia, targeting their core enzyme, the isolated strain exhibited unusually high tolerance. As the interplay between methane oxidation and ammonia is thought to play a key role in natural ecosystems (14, 15), discovery of this unique feature changes how we understand the ecology of methane cycling. Additionally, the strain showed methanotrophy in a wider pH range than conventional methanotrophs. The tolerance of ammonia and a wide pH range may allow mycobacterial methanotrophs to fill niches where conventional methanotrophs are challenged.

## MATERIALS AND METHODS

### Reactor configurations and operational conditions

Two identical down-flow hanging sponge (DHS) reactors (Fig. S1) were employed for the enrichment of methanotrophs. Each reactor was composed of a 140 mL glass column measuring 200 mm in height and 30 mm in diameter (Fig. S1). Within each column, a series of nine polyurethane sponge cubes (each cube of 1 cm³) was diagonally suspended on a nylon string within the gas phase. Prior to setting up the DHS reactors, the sponge carriers were immersed in activated sludge obtained from an aeration tank at a municipal wastewater treatment plant in Higashihiroshima, Japan, to serve as the inoculum. The DHS reactors were operated under two different NH_4_^+^ concentrations: 143 mM (referred to as reactor HR) and 0.14 mM (referred to as reactor LR). The composition of the medium was as follows (L^−1^): 1 mL of mineral solution, 1 mL of trace element solution, 1 mL of FeSO_4_·7H_2_O solution, 1 mL of KNO_3_ solution, 1 mL of KH_2_PO_4_ solution, and NH_4_Cl adjusted to the planned NH_4_^+^ concentration (Table S1). The pH was maintained at 4 through the addition of H_2_SO_4_ to avoid enrichment of ammonia-oxidizing bacteria, which would theoretically compete with methanotrophs for oxygen. An air mixture comprising 10% methane as the energy substrate was continuously supplied to the reactors via a peristaltic pump. To minimize reactor longitudinal gradients in methane and NH_4_^+^ concentrations, the effluents were recirculated at 10 times the influent flow rate. Both reactors were maintained within a temperature-controlled chamber at 25°C.

### Chemical analysis

Methane concentration was quantified utilizing a gas chromatograph equipped with a thermal conductivity detector (GC-8A; Shimadzu, Kyoto, Japan). To determine NH_4_^+^ concentration, an ion chromatograph (HPLC-20A, Shimadzu) was employed. Concentrations of NO_2_^–^ were measured by high-performance liquid chromatography using a TSKgel SAX column (Tosoh Co., Shunan, Japan) and a UV-VIS detector (GL-7451; GL Science, Tokyo, Japan). The pH of liquid solutions was assessed using a pH meter (pH meter F-52; HORIBA, Kyoto, Japan).

### Microbial community analysis of the bioreactor via 16S rRNA gene sequencing

DNA was extracted using the FastDNA SPIN Kit for Soil (MP Biomedicals, California, USA). The V3–V4 hypervariable regions of the 16S rRNA gene were PCR-amplified using the primer pair 341′f (5′-CCTACGGGNGGCWGCAG-3′) and 805r (5′-GACTACHVGGGTATCTAATCC-3′), employing TaKaRa Ex Taq Hot Start Version (Takara Bio, Shiga, Japan). The PCR protocol followed was as per previously established conditions (16). Purification of the PCR products was performed using Agencourt AMPure XP (Beckman Coulter, California, USA), and subsequent sequencing was conducted on the Illumina Miseq platform with paired-end sequencing (300 bp × 2) using a Miseq reagent kit (v.3; Illumina, California, USA) at Bioengineering Lab. Co., Ltd. (Kanagawa, Japan). Sequence processing involved primer trimming and quality filtering with Cutadapt (v.1.18) (17) and Trimmomatic (v.0.39-1) (18), respectively. Cleaned sequences were merged using fastq–join (v.1.3.1) (19). For downstream analyses, the merged sequence reads were processed utilizing QIIME 2 core 2022.2 (20, 21), as previously detailed (22). Taxonomic assignment was performed with the Silva 138.1 database (23).

### Plate and liquid culture preparation

The mineral composition and concentrations were consistent with those used in the reactors, and the pH was set at 4. In the case of plate cultures, a 2.2% gellan gum medium was utilized. Inoculated plates were enclosed within Anaero Pack resin bags (Mitsubishi Gas Chemical, Japan), supplied with an air mixture containing 10% methane for cultivation. In liquid cultures, biomass samples were introduced into glass vials (Nichiden-Rika Glass Co., Ltd., Japan) containing the medium (a ratio of 1:2 for the liquid phase and the gas phase). After sealing the vials with butyl rubber stoppers (Maruemu Corporation, Japan) and aluminum crimps (Maruemu Corporation), methane concentration was set at 10% by injecting methane into the gas phase through a 0.2 µm filter.

### Isolation and verification of purity

Liquid samples from the *Mycobacterium*-enriched bioreactor community were plated on solid media, and colonies were further sub-cultured in liquid media. This was repeated three times to obtain a pure culture of the target strain. To verify the purity of the isolated strain, we microscopically checked consistency in cell morphology (Olympus BX53, Japan), and conducted 16S rRNA gene amplicon sequencing, shotgun genome sequencing, and cultivation with complex organic substrates as described below.

For 16S rRNA gene amplicon sequencing, DNA was extracted from cultures of MM-1 using the proposed method by Epperson and Strong (24). A barcoded 16S rRNA gene amplicon library was prepared using primer sets as described in Table S2, employing TaKaRa La Taq (Takara Bio). PCR was performed with an initial denaturation at 96°C for 1 min, followed by 35 cycles at 96°C for 25 s, at 52°C for 45 s, and at 72°C for 1 min; final extension was performed at 72°C for 7 min. Purification of the PCR products and subsequent sequencing were performed at our laboratory in the same manner as described above (see “Microbial community analysis of the bioreactor via 16S rRNA gene sequencing”). Sequence processing involved merging with pear (v.0.9.10) (25), primer trimming, and quality filtering with Cutadapt (v.4.1) (17), respectively. The merged sequence reads were filtered using a custom Perl script with the following criteria: more than 98% of the bases had a quality score of 30 or higher; the minimum sequence length was set to 100 bases; and the maximum length was 550 bases. For downstream analyses, the merged sequence reads were processed utilizing QIIME 2 core 2024.5 (20, 21). Taxonomic assignment was performed with the Silva 138.1 database (23).

For genome shotgun sequencing (see “Genome sequencing, assembly, and annotation,” below), the reads cleaned by Trimmomatic (LEADING: 20 TRAILING: 20 SLIDINGWINDOW: 4:20 MINILEN: 100) were mapped to the whole genome of MM-1 using Bowtie2 (v.2.3.5.1) (26) with the “--end-to-end” option.

To verify the purity of the culture, we implemented 16S rRNA amplicon sequencing and shotgun genome sequencing. Given that both sequencing approaches are prone to contamination (27) (e.g., from sample bleeding [28], index hopping [29], and index switching [30] for amplicon sequencing), differentiation of organisms that are present in the culture and those whose sequences appear due to technical contamination is critical. First, sequences that comprise less than 0.3% of the amplicon sequencing results were considered contamination that was introduced during sequencing (27). Second, we compared the phylogeny of sequences that were not assigned to MM-1 between the amplicon and shotgun genome sequencing results, and taxa that only appear in one of the data sets were also considered sequencing-based contamination.

In previous studies on proteobacterial methanotrophs that are obligately methanotrophic/methylotrophic, culture purity is often checked by plating on solid complex organic media (i.e*.*, verify absence of organotrophic microorganisms). Here, we used R2A (DSMZ830) and LB (DSMZ381) medium to check whether MM-1 can grow on complex organics and, if not, to verify the absence of contaminating organotrophic bacteria that were not detectable by the above sequencing. If colonies had appeared, we would have conducted direct sequencing to determine whether they were contaminations. Since no colonies appeared in this study, sequencing was not performed. Plates were cultivated at 30°C for 3 months.

### Scanning electron microscopy

Microbial cells were fixed in 2.5% (wt/vol) glutaraldehyde in the liquid medium at 30 °C for 2 h. The sample preparation procedure has been described previously (31). The cell samples were observed using extreme high-resolution focused ion beam scanning electron microscopy (Helios G4 UX; Thermo Fisher Scientific, USA).

### Methane consumption and optical density measured at 600 nm

The methane consumption and optical density measured at 600 nm (OD_600_) of MM-1 were quantified using cultures grown on a liquid medium. Five milliliters of MM-1 liquid culture cultivated for 1–2 months with a headspace containing 10% methane was inoculated into autoclaved 50 mL vials (effective volume of 70 mL; Nichiden-Rika Co., Ltd.) containing fresh medium (20 mL). The vials were sealed using butyl rubber stoppers (Maruemu Corporation) and aluminum crimps (Maruemu Corporation). Methane was then injected into the headspace through a 0.2 µm filter. Methane in the headspace was measured by collecting 0.2 mL of headspace and analyzing this using gas chromatography as described above. OD_600_ was also measured using a JASCO V-650 spectrometer (JASCO Corporation, Japan). Incubations were performed in triplicate.

### Physiological characterization

The methane oxidation capability of MM-1 was investigated using a liquid medium at different temperatures (4°C–45°C), NH_4_^+^ (1.43–500.0 mM), and pH (0.5–10.0) conditions. The bacterial biomass, obtained from 150 mL of liquid culture, was divided equally into tubes and washed with the medium corresponding to NH_4_^+^ and pH conditions. After transferring each biomass into 10 mL vials (effective volume of 15 mL; Nichiden Rika Glass Co., Ltd.), liquid medium was added to adjust the total volume to 10 mL before sealing the vials with butyl rubber stoppers (Maruemu Corporation) and aluminum crimps (Maruemu Corporation). Methane was then injected into the gas phase through a 0.2 µm filter. A 0.2 mL gas sample from the gas phase was periodically collected and analyzed using gas chromatography. All incubations were performed in triplicate.

To determine the substrate usage, the following substrates were used: methanol (0.05%, vol/vol), formate (0.05% of sodium formate, wt/vol), 1-propanol (0.05%, vol/vol), pyruvate (1 mM), succinate (1 mM), malate (1 mM), acetate (1 mM), glucose (1 mM), fructose (1 mM), xylose (1 mM), arabinose (1 mM), ribose (1 mM), galactose (1 mM), sucrose (1 mM), maltose (1 mM), and yeast extract (0.1%, wt/vol) in the same medium as the reactors. All the cultivation for the substrate test was performed on plates in duplicate, and the plates were monitored. The observation period was extended to approximately 5 months, which is roughly four times the standard period for colony appearance of the isolated strain on methane, thereby exceeding the duration typically required by conventional methods (32). Substrate utilization was assessed based on the appearance of colonies, and the 16S rRNA gene of the resulting colonies was identified by direct sequencing. The utilization of MM-1 on methanol (0.05%, vol/vol) and formate (0.01%, 0.05%, and 0.1% of sodium formate, wt/vol) was also investigated using liquid culture. Growth was confirmed by quantitative polymerase chain reaction (qPCR) as described below.

### qPCR assay for MM-1

For DNA extraction, cell lysis was conducted using the method proposed by Epperson and Strong (24), with minor modifications as described below. Cell pellets were collected from 1 mL of liquid culture and resuspended in 50 µL of lysis buffer. Then, 12.5 µL of lysozyme was added to the suspension. The sample was incubated at 37°C for 10 min. Subsequently, 12.5 µL of 2.5 mg/mL proteinase K and 25 µL of 20% SDS were added, and the sample was incubated at 65°C for 10 min. We confirmed that the DNA yield was consistent regardless of incubation time variations. Following cell lysis, 30 µL of 3M potassium acetate (pH 4.8) was added to the sample and incubated on ice for 5 min. The supernatant was carefully removed, and the DNA was precipitated using isopropanol. The precipitated DNA was washed with 75% ethanol, dried using an evaporator, and resuspended in DNase and RNase-Free TE buffer (Nippon Gene, Japan). DNA concentrations were measured using Qubit 4.0 fluorometer (Thermo Fisher Scientific).

For qPCR, template DNA was prepared from either the original or diluted DNA samples. Primer set of B27F (5′-AGAGTTTGATCCTGGCTCAG-3′)/Myco179R (5′-TGCGTCCCGAGATCCTAT-3′) was used. The primer Myco179R specific to the 16S rRNA gene sequence of MM-1 was designed using the ARB program (33). The PCR condition was as follows: initial denaturation at 95°C for 30 s, followed by 40 cycles of denaturation at 95°C for 10 s, annealing at 56°C for 30 s, and extension at 72°C 30 s. The annealing temperature was optimized empirically through the amplification of the 16S rRNA gene of MM-1. To verify the specificity of the qPCR assay, a melting-curve analysis was performed for every qPCR assay.

### Genome sequencing, assembly, and annotation

High-molecular-weight genomic DNA was extracted from cultures of MM-1 using the proposed method by Epperson and Strong (24). The genome of MM-1 was sequenced at GeneBay, Inc. (Kanagawa, Japan) using the PromethION (long-read sequencing) (Oxford Nanopore Technology, Oxford, UK) and at Bioengineering Lab. Co., Ltd. using DNBSEQ-G400 (200 bp × 2) (MGI Tech Co., Ltd., Shenzhen, China) platforms. The Nanopore reads were processed using a quality threshold of 15 and a length threshold of 10 kbp. When assembled using Flye 2.9.2-b1786 (34), a single contig was constructed, and the circularity was confirmed by reads bridging the two ends of the contig. The consensus contig was polished using medaka (v.1.11.3) (https://github.com/nanoporetech/medaka) and Polypolish (v.0.6.0) (35). Open reading frames were predicted using Prodigal (v.2.6.3) (36). DIAMOND (v.2.0.14) and eggNOG-mapper were employed to search for homologous enzymes (37–39). Additionally, a comprehensive investigation was conducted across methanotrophs in *Actinomycetota*, *Pseudomonadota*, *Verrucomicrobiota*, and *Ca*. Methylomirabilota, confirming the presence or absence of genes related to methane oxidation (*pmoA* and *mmoX*) and those likely involved in NH_4_^+^/NH_3_ inhibition of methanotrophs (*hao*, ammonium transporter gene, and nitrate/nitrite transporter gene). The eggNOG-mapper tool assigned KO numbers to the methanotrophs, as detailed in Table S3.

### Phylogenetic analysis

A maximum-likelihood tree based on 16S rRNA gene sequences was constructed using representative sequences collected from GenBank under the family *Mycobacteriaceae*. Sequences were aligned using MAFFT (v.7.48) (40) (--maxiterate 1000—localpair). A maximum-likelihood tree was calculated using IQTREE (v.2.2.6) (41) via an optimal model chosen by ModelFinder (-m MFP) and 1,000 ultrafast bootstrap replicates (-bb 1000). Bootstrap values were recalculated using BOOSTER (v.0.1.2) (42).

We also performed maximum-likelihood estimation of a phylogenetic tree using a set of marker proteins conserved among most bacteria defined in GTDB r214 (43). Species representative genomes in GTDB r214 were collected for the genus *Mycobacterium* and several representatives of closely related genera in the family *Mycobacteriaceae*. Marker proteins were aligned using MAFFT (--maxiterate 1000—localpair), concatenated, and trimmed using BMGE (v.1.12) (44) with the BLOSUM30 matrix and maximum gap rate of each position of 0.67 and minimum length of selected regions of 3. Maximum-likelihood estimation was performed using IQ-TREE (v.2.2.6) (41) with the universal distribution mixture model with 64 components and log-centered log-ratio transformation and 1,000 ultrafast bootstrap replicates (-B 1000). Bootstrap values were recalculated using BOOSTER.

The phylogeny of *mmoX* was analyzed by collecting *mmoX* sequences (Kyoto Encyclopedia of Genes and Genomes Orthology K16157) from GTDB r214 using AnnoTree, clustering with a sequence identity threshold of 90% using CD-HIT (v.4.8.1), alignment using MAFFT (--maxiterate 1000—localpair), trimming using trimAl (v.1.4) (-keepseqs -gt 0), and maximum-likelihood estimation of the phylogeny using IQ-TREE (v.2.3.6) (41) with a model of LG + G4 and 1,000 ultrafast bootstrap replicates (-B 1000).

### RNA-based sequencing analysis

To confirm the expression of genes in MM-1, RNA-based sequencing analysis was conducted. MM-1 was incubated with CH_4_ at 14.3 mM NH_4_^+^ at 30°C in liquid culture. After incubation periods of 12 and 27 days, respectively, 100 mL of the culture liquid samples was collected from each vial for analysis. Total RNA was extracted from microbial cells and concentrated using a Direct-zol RNA Miniprep according to the manufacturer’s instructions, with inclusion of an additional step of homogenization using ZR BashinBead Lysis Tubes (Zymo Research, USA) after treatment with TRI Reagent. For rRNA removal from the extracted RNA, we used RiboCop rRNA Depletion Kit for Mixed Bacterial Samples (META) (Lexogen GmbH, Austria). RNA-Seq libraries were constructed by complementary DNA synthesis using the SMARTer Stranded RNA-Seq Kit (Takara Bio USA, Inc., California, USA). The libraries obtained were sequenced using the MiSeq platform (Illumina), yielding 3,971,261 and 4,110,139 paired-end sequences (300 bp × 2) for the day 12 and 27 samples. Raw sequences were trimmed using Trimmomatic (LEADING: 20 TRAILING: 20 SLIDINGWINDOW: 4:20 MINILEN: 100). Gene expression values of MM-1 were calculated using Rockhopper (v.2.03) (allowed mismatches: 0.05) (https://cs.wellesley.edu/~btjaden/Rockhopper/).

### 16S rRNA marker-based survey of published amplicon data

To investigate the distribution of MM-1 and *Ca*. Mycobacterium methanotrophicum, a 16S rRNA marker-based survey of published amplicon data was conducted using Integrated Microbial Next Generation Sequencing (https://www.imngs.org/) for hits with more than 97% identity and 150 bp (45).

## RESULTS AND DISCUSSION

Two DHS reactors inoculated with activated sludge were operated with either a low concentration of NH_4_^+^ (0.14 mM, LR) or a concentration high enough to inhibit all known methanotrophs (143 mM, HR), both at pH 4.0. Despite the inhibitory conditions in HR, we observed clear methane consumption, albeit starting much later than LR (Fig. S2 and S3). While LR contained a known methanotroph genus, *Methylocystis* of *Pseudomonadota* (9.1%), HR lacked any known methanotrophic lineages and had a highly enriched population of *Mycobacteriaceae* (29.0%, amplicon sequence variant [ASV] 1–4), which was also detected in LR (8.5%) (Fig. S4 and Note S1). These reactor operations confirmed that high NH_4_^+^ concentrations inhibit conventional methanotrophs detectable by 16S rRNA genes sequencing.

**Fig 1:**
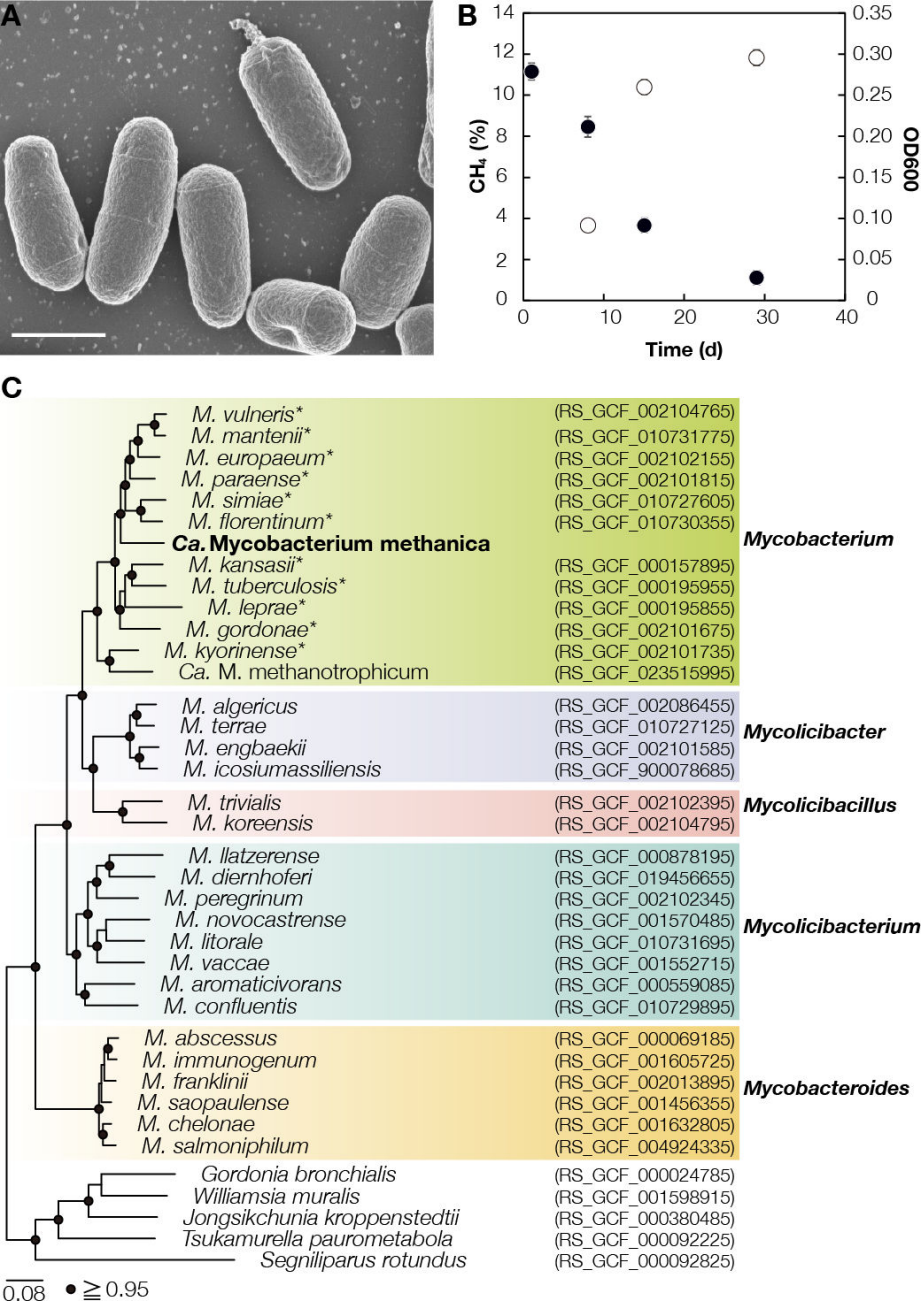
Morphology, methane oxidation activity, and phylogeny of Mycobacterium methanica MM-1. (A) Photomicrograph of strain MM-1 cultured using methane at 14.3 mM of NH_4_^+^ under scanning electron microscopy. Bar represents 1 µm. (B) Methane consumption at 14.3 mM of NH_4_^+^ by MM-1 and optical density measured at 600 nm (OD_600_). The filled and open circles represent the methane concentration and OD_600_, respectively (*n* = 3). (C) Phylogenetic tree of a subset of species from the family *Mycobacteriaceae* based on a concatenated alignment of single-copy marker genes included in GTDB r214. *Indicates *Mycobacterium* spp. containing pathogens.

To isolate the putatively highly NH_4_^+^-tolerant and phylogenetically novel mycobacterial methanotroph, we inoculated HR biomass on solid medium under a methane-air mixture (1:9, vol/vol). We selected 100 colonies for inoculation in liquid media with 143 mM NH_4_^+^ to identify colonies exhibiting NH_4_^+^-tolerant methanotrophy. Approximately 20 out of 100 vials showed turbidity and methane consumption, most of which contained the *Mycobacterium* population. For downstream isolation, we performed additional alternating cultivation in high-NH_4_^+^ (143 mM) liquid media and solid media with a less inhibitory NH_4_^+^ concentration (7.1 mM). Subsequent incubations used a liquid medium with high NH_4_^+^ (143 mM) to prevent conventional methanotroph contamination. (The high-NH_4_^+^ condition was used to prevent growth of conventional methanotrophs, and the lower NH_4_^+^ concentration was used to accelerate growth of the target methanotroph.) This led to the isolation of a novel methanotroph strain, MM-1, of nonmotile and rod-shaped cells, which demonstrated the methane consumption with an increase in optical density at 600 nm (Fig. 1A and B). The isolated strain typically forms colonies within 1.5–2.0 months with methane as a substrate (Fig. S5). Culture purity was verified through (i) microscopy (Fig. S6); (ii) Illumina sequencing of a 16S rRNA gene amplicon; (iii) mapping of shotgun short reads onto the genome; and (iv) cultivation on plates with complex organic media for detection of any heterotrophs, if present (see Materials and Methods for details).

Growth on substrates other than methane was also tested using basal solid or liquid media supplemented with each substrate. MM-1 was able to grow on methanol but did not exhibit growth on formate in liquid culture (Fig. S7). While colonies of MM-1 could form on plates supplemented with acetate, succinate, glucose, or maltose, such growth was not consistently replicable, and colonies emerged after 5 months of cultivation. At this point, we cannot exclude the possibility that they grew using impurities in the solid medium. Thus, future studies are necessary to explore organotrophic/heterotrophic growth by MM-1.

**Fig 2:**
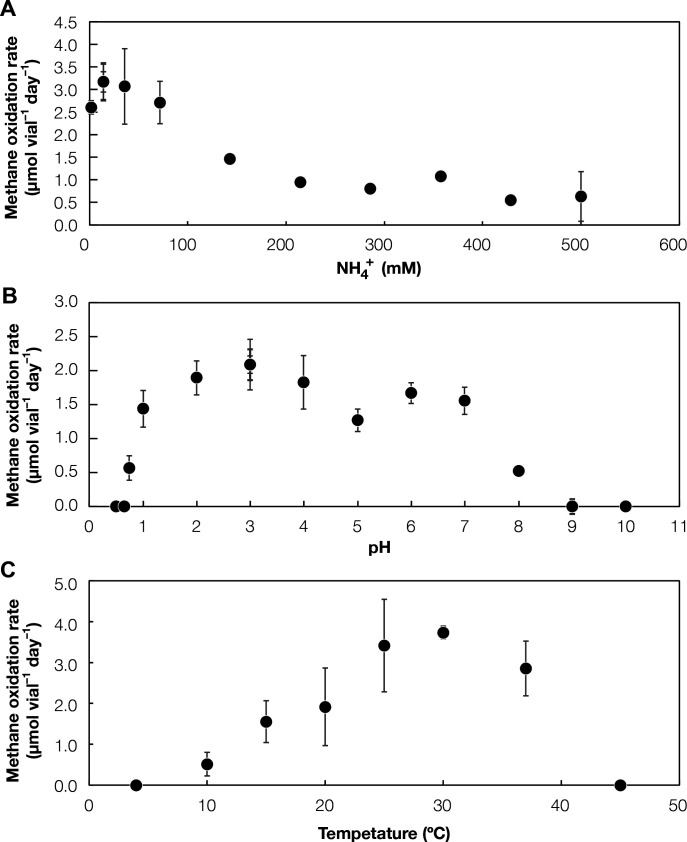
Physiological traits of Mycobacterium methanica MM-1. (A) The effect of NH_4_^+^ on methane oxidation activity at pH 4 and 30°C (*n* = 3). (B) The effect of pH on methane oxidation activity at 14.3 mM of NH_4_^+^ and 30°C. (*n* = 3). (C) The effect of temperature on methane oxidation activity at pH 4 and 14.3 mM of NH_4_^+^ (*n* = 3).

The strain could oxidize methane in a temperature range of 10°C–37°C (Fig. 2C). This isolate belongs to the phylum *Actinomycetota*, with a 16S rRNA gene sequence identical to the dominating mycobacterial population in HR (ASV1) (Fig. S8). Phylogenetic analyses of both single-copy marker genes and the 16S rRNA gene placed MM-1 in the genus *Mycobacterium* predominated by species containing pathogenic isolates (Fig. 1C; Fig. S8).

MM-1 only possesses a soluble methane monooxygenase (sMMO), similar to *Ca*. M. methanotrophicum (13), and some proteobacterial methanotrophs: *Methylocella*, two strains from *Methyloferula* (46), and *Methyloceanibacter* (47). Phylogenetic comparison of *mmoX* sequences between mycobacterial and proteobacterial methanotrophs reveals that mycobacterial *mmoX* forms a clearly distinct phylogenetic group from other methanotrophic bacteria (Fig. S9). While MM-1 has the capacity to oxidize methanol as described above (Fig. S7), it lacks genes encoding pyrroloquinoline quinone-dependent methanol dehydrogenase, which is present in all other known methanotrophic isolates. Instead, we identify high expression of an alcohol dehydrogenase gene in methane-fed MM-1 cultures (Table S5). The same was observed for *Ca*. M. methanotrophicum (13). This indicates that the energy metabolism of mycobacterial methanotrophs differs from conventional methanotrophic bacteria. Subsequent oxidation of formaldehyde and formate is conducted by glutathione-independent formaldehyde dehydrogenase and formate dehydrogenase, respectively, like conventional methanotrophs. Formaldehyde can also be assimilated by MM-1 via the ribulose monophosphate cycle, a pathway commonly observed for *Pseudomonadota* methanotrophs. The genes related to the serine pathway were not identified in the genome. Transcriptomics verified high expression of genes encoding proteins involved in methanotrophy, as described above (Table S5). MM-1 also possesses the Calvin-Benson-Bassham cycle as another carbon fixation pathway, which is also found in *Verrucomicrobiota* (48), NC10 (49), and some *Pseudomonadota* methanotrophs (50). These pathways for methanotrophy and autotrophy are found in both MM-1 and *Ca*. M. methanotrophicum.

**Fig 3:**
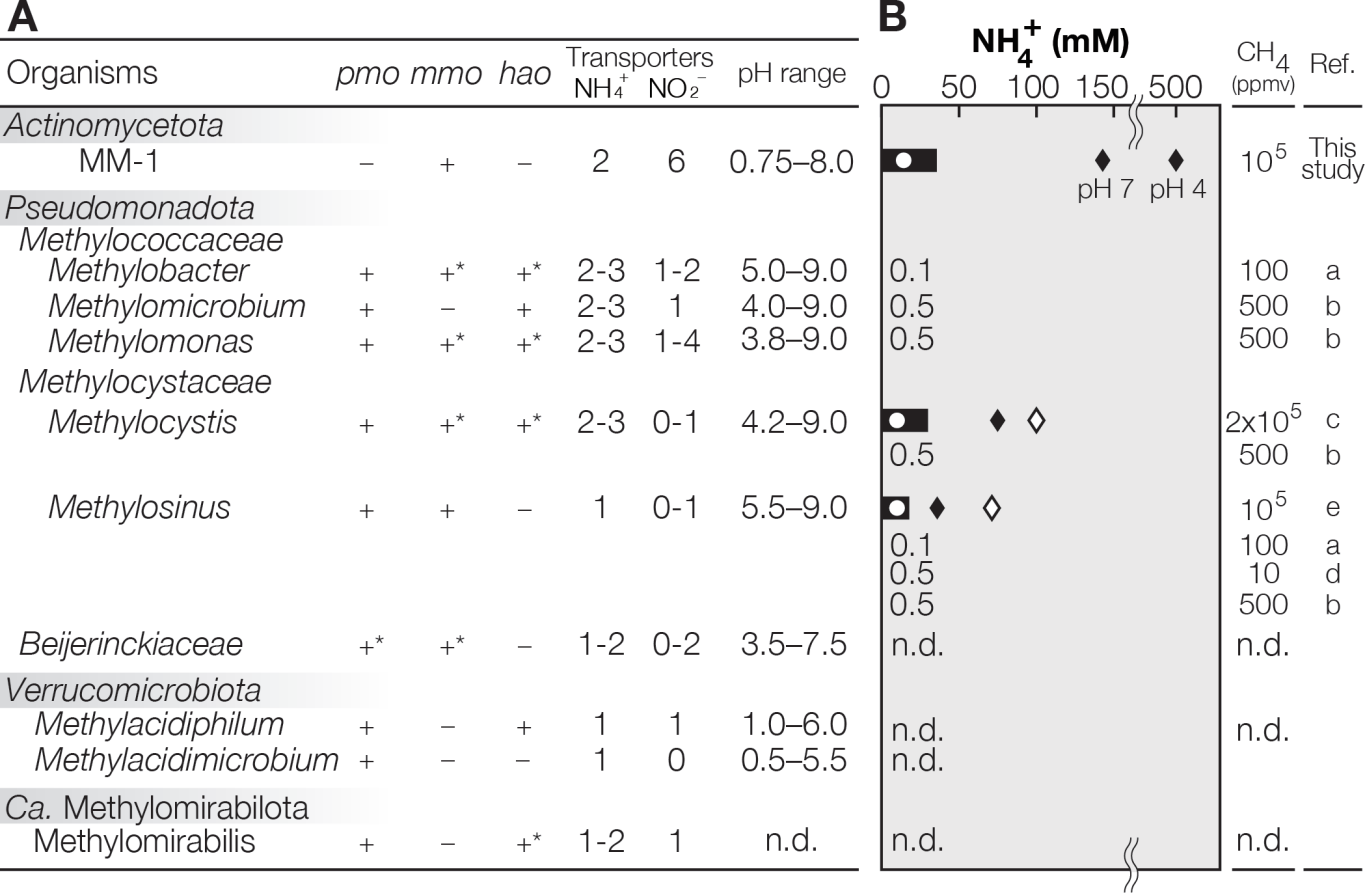
Comparison of genetic and phenotypic characteristics of MM-1 and known methanotrophs. (A) List of genes for methane oxidation (*pmo* and *mmo* genes, encoding pMMO and sMMO, respectively) and for those likely related to dealing with the presence of high levels of NH_4_^+^ (*hao* encoding hydroxylamine oxidoreductase, ammonium transporter genes, and nitrate/nitrite transporter genes), along with pH optima and pH range. +, +^*^, and − indicate the presence, the mixture of the presence and absence, and the absence of the genes, respectively. KO number corresponding to each gene: K10944 (*pmoA*), K16157 (*mmoX*), K10535 (*hao*), K03320 (ammonium transporter genes), and K02575 (nitrate/nitrite transporter genes). The details on information of each species are given in Table S3. (B) NH_4_^+^ concentrations tested for NH_4_^+^/NH_3_ inhibition of MM-1 and, for comparison, reported concentrations for other methanotrophic isolates. The bar and values indicate the minimum inhibitory concentration. Open circles indicate the highest tested concentrations at which no inhibition was observed. Filled diamonds indicate the maximum concentrations at which methanotrophic activity was detected, and open diamonds indicate the lowest concentrations at which methanotrophic activity was lost. The experiments for MM-1 were conducted at pH 4 and 7. The reported ammonia inhibition experiments were performed at a neutral pH (6.8 or 7.0). a, King and Schnell (54); b, Nyerges and Stein (55); c, Guo et al. (52); d, King and Schnell (56); e, He et al. (51).

MM-1 showed methane-oxidizing capacity up to 500 mM NH_4_^+^ at pH 4 (Fig. 2A). The highest oxidation rate occurred at 14.3 mM NH_4_^+^ and inhibition only began at 35.5 mM NH_4_^+^ (Fig. S10), which is much higher than the minimum inhibitory concentrations of other methanotrophs in the phylum of *Pseudomonadota* (Fig. 3B). MM-1 could still retain high methane-oxidizing activity at 143 mM, a concentration at which conventional methanotrophs are fully inhibited (51, 52). Given that we also detected *Mycobacterium* in reactors operated at higher pH (pH 7) (16), we also investigated whether the strain exhibits this tolerance under neutral conditions. The strain showed the same level of tolerance at pH 7 based on the comparable methane oxidation rates (Fig. S10). This was unexpected as the theoretical concentration of NH_3_—the inhibitory form of NH_4_^+^—is approximately 1,000 times greater at pH 7 than at pH 4. Other verrucomicrobial methanotrophs may also have high ammonia tolerance, but this has yet to be quantitatively investigated (53).

Strain MM-1’s heightened tolerance of NH_3_ may stem from the cellular location of the enzyme responsible for methane oxidation: methane monooxygenase (MMO). As described above, MMOs are categorized into two variants: pMMO, a membrane-bound form, and sMMO, a cytoplasmic form. Given that pMMO operates outside the cytoplasm, it is likely more challenging for the cell to protect pMMO from high environmental concentrations of NH_3_ (e.g., via transporter-mediated NH_4_^+^ export) than cytosolic sMMO. Thus, MM-1’s exclusive use of sMMO may make its methane metabolism less susceptible to NH_3_ inhibition than most other methanotrophs, which possess pMMO (Fig. 3A ; Table S3).

Hydroxylamine (NH_2_OH), which is an inadvertent product of NH_3_ oxidation by MMOs, also functions as a methanotrophy inhibitor; thus, most known methanotrophs encode hydroxylamine oxidoreductases (HAOs) to decompose NH_2_OH to nitric oxide (57) and, ultimately, nitrite (NO_2_^–^) (52). However, MM-1’s genome lacks HAO homologs (Fig. 3A). Some methanotrophs with pMMO in the phyla *Pseudomonadota* and *Verrucomicrobiota* also lack HAO (Table S3), and two possibilities for ammonia tolerance mechanisms have been proposed (58): (i) inability of their MMO to oxidize ammonia or (ii) hydroxylamine degradation via an unidentified protein/pathway. In MM-1, NO₂⁻ production was detected during cultivation at pH 7, where NH_3_ concentrations are higher (Fig. S10). These findings indicate the presence of an unidentified enzyme mechanism for converting NH₂OH to NO₂⁻.

Methanotrophy has been shown to be negatively influenced by exposure to ammonia in various environments, including lakes, agricultural soils, and landfills (14, 15, 59, 60). Ammonia addition reduced methane uptake/consumption by approximately 30%–50% (15, 59), and the inhibition persisted over months to years (61, 62). Thus, the fate of methane in natural environments would largely depend on the ammonia tolerance of indigenous methanotrophs. In theory, the ratio of ammonia to methane (NH_3_:CH_4_) may play a major role in the degree of inhibition, as ammonia acts as a competitive inhibitor of methane oxidation. Conventional methanotrophs begin to experience inhibition at NH_3_:CH_4_ ratio of approximately 0.4–0.5 and cease activity at 1.4–2.0, as calculated using data from Guo et al. (52) and He et al. (51). Meanwhile, MM-1 began to show inhibition at 1.4 and maintained the activity even at 6.5 at pH 7.0, suggesting that MM-1 has an advantage in methanotrophy in environments with NH_3_/CH_4_ exceeding 0.4–0.5 (e.g., surface waters downstream of wastewater treatment plants) (63). For reference, even in environments with low ammonia concentrations (e.g., ≤1 mg/L like drinking water [64]), the NH_3_/CH_4_ ratio can theoretically be as high as 132.9, assuming the dissolved methane is in equilibrium with atmospheric methane. *Mycobacterium*’s (and potentially *Actinomycetota*’s) significant resistance to ammonia would allow them to serve as methane sinks in various settings where conventional methanotrophs would be challenged, which may play key roles in mitigating methane emissions from natural ecosystems to the atmosphere.

Another important characteristic of MM-1 is the high adaptability of *Mycobacterium* methanotrophy to a wide range of pH (Fig. 2B). This isolate remarkably demonstrated methane oxidation capacity across a pH range broader than any previously reported methanotroph (Fig. 3A ; Table S3): pH of 0.75–8.0. The genus *Mycobacterium* includes pathogenic species, which may have evolved to tolerate a wide pH range as an adaptation to varying pH environments encountered inside host (65). The optimum pH was approximately 3–4, and *Ca.* M. methanotrophicum was frequently detected in environments with a pH of around 4 (13), suggesting that methanotrophic *Mycobacterium* may favor acidic conditions. While verrucomicrobial methanotrophs can also thrive in extremely acidic conditions, they are far thought to strictly inhabit high-temperature environments. In total, MM-1 greatly expands our understanding of the habitable environmental range. Reflecting this newly discovered methanotroph from the phyla of *Actinomycetota*, we propose the following name for the strain, *Ca*. Mycobacterium methanica (Note S2). (Note that “*Candidatus*” should be prepended, given that validation of the name has not been completed.)

We performed a 16S rRNA gene-based survey of published amplicon data and detected methanotrophic *Mycobacterium* spp. (≥97% or 99% sequence identity) in methane-containing environments, including natural (e.g., soils and lakes), host-associated (fish/insect gut and plants), and artificial (e.g., drinking water distribution systems and activated sludge) environments (Table S4). Notably, the relationship between drinking water distribution systems and methanotrophs has recently attracted concern in the environmental engineering and health field (66). The potential for methane to support growth of *Mycobacterium* spp. and their innate ability to theoretically tolerate the relatively high ammonia-to-methane concentration ratio *in situ* (67) shed new insight on potential ecological relationships between methane and pathogens in engineered systems.

Overall, M. methanica MM-1 not only represents the first isolate of *Actinomycetota* methanotrophs but also reveals methanotrophy physiologically distinct from conventional methanotrophs. These suggest the presence of overlooked methane sinks and overturn the preconception that methanotrophs are easily influenced by ammonia. The significance of *Mycobacterium*, so far strongly associated with the medical field for pathogenicity, certainly extends to the natural environment and may connect both fields, thus highlighting the need for future studies on the evolutionary path to the emergence of this niche. Furthermore, for comprehensive evaluation of methanotrophy’s contributions to the global methane cycle, future research should delve into the investigation of mechanisms supporting the extraordinary ammonia tolerance of *Mycobacterium*, other methanotrophic *Actinomycetota* spp., and the ecological contribution of the ammonia-tolerating versatile methanotrophy to the global carbon cycle.

## Data Availability

The 16S rRNA gene amplicon sequences obtained from bioreactor enrichments and MM-1 liquid culture purified have been deposited at the National Center for Biotechnology Information (NCBI) under accession numbers DRR533648-DRR533651 and DRR686848. The genome for Mycobacterium methanica MM-1 is available under GenBank BioProject accession number PRJDB17603. Raw sequencing data of the MM-1 transcriptome have been deposited in the NCBI Sequence Read Archive database under the accession number DRR553409-DRR553412.

## SUPPLEMENTAL MATERIAL

Supplemental material, part I aem.00796-25-s0001.docx Fig. S1 to S10, Tables S1 and S2, and Notes S1 and S2.

Supplemental material, part II aem.00796-25-s0002.xlsx Tables S3 to S5.

ASM does not own the copyrights to Supplemental Material that may be linked to, or accessed through, an article. The authors have granted ASM a non-exclusive, world-wide license to publish the Supplemental Material files. Please contact the corresponding author directly for reuse.

## References

[1] Intergovernmental Panel on Climate Change (IPCC). 2023. The earth’s energy budget, climate feedbacks and climate sensitivity, p 923–1054. In Climate change 2021 – The physical science basis: Working Group I contribution to the sixth assessment report of the intergovernmental panel on climate change. Cambridge University Press, Cambridge.

[2] Lan X, Thoning KW, Dlugokencky EJ. 2025. Trends in globally-averaged CH4, N2O, and SF6 determined from NOAA global monitoring laboratory measurements. Version. doi:10.15138/P8XG-AA10

[3] Canadell JG, Monteiro PMS, Costa MH, Cunha LCD, Cox PM, Eliseev AV, Henson S, Ishii M, Jaccard S, Koven C, et al.. 2021. Global carbon and other biogeochemical cycles and feedbacks. In Final government distribution

[4] Stevenson DS, Zhao A, Naik V, O’Connor FM, Tilmes S, Zeng G, Murray LT, Collins WJ, Griffiths PT, Shim S, Horowitz LW, Sentman LT, Emmons L. 2020. Trends in global tropospheric hydroxyl radical and methane lifetime since 1850 from AerChemMIP. Atmos Chem Phys 20:12905–12920. doi:10.5194/acp-20-12905-2020

[5] Hanson RS, Hanson TE. 1996. Methanotrophic bacteria. Microbiol Rev 60:439–471. doi:10.1128/mr.60.2.439-471.1996 8801441 PMC239451

[6] Conrad R. 2009. The global methane cycle: recent advances in understanding the microbial processes involved. Environ Microbiol Rep 1:285–292. doi:10.1111/j.1758-2229.2009.00038.x 23765881

[7] Pol A, Heijmans K, Harhangi HR, Tedesco D, Jetten MSM, Op den Camp HJM. 2007. Methanotrophy below pH 1 by a new Verrucomicrobia species. Nature 450:874–878. doi:10.1038/nature06222 18004305

[8] Ettwig KF, Butler MK, Le Paslier D, Pelletier E, Mangenot S, Kuypers MMM, Schreiber F, Dutilh BE, Zedelius J, de Beer D, Gloerich J, Wessels HJCT, van Alen T, Luesken F, Wu ML, van de Pas-Schoonen KT, Op den Camp HJM, Janssen-Megens EM, Francoijs K-J, Stunnenberg H, Weissenbach J, Jetten MSM, Strous M. 2010. Nitrite-driven anaerobic methane oxidation by oxygenic bacteria. Nature 464:543–548. doi:10.1038/nature08883 20336137

[9] Bay SK, Dong X, Bradley JA, Leung PM, Grinter R, Jirapanjawat T, Arndt SK, Cook PLM, LaRowe DE, Nauer PA, Chiri E, Greening C. 2021. Trace gas oxidizers are widespread and active members of soil microbial communities. Nat Microbiol 6:246–256. doi:10.1038/s41564-020-00811-w 33398096

[10] Kozlova EI, Vorob’eva LI, Arkad’eva ZA, LIaDYG R. 1969. Microorganisms oxidizing methane. Mikrobiologiya 38:251–257. 4899392

[11] Reed WM, Dugan PR. 1987. Isolation and characterization of the facultative methylotroph Mycobacterium ID-Y. J Gen Microbiol 133:1389–1395. doi:10.1099/00221287-133-5-1389 3655742

[12] NECHAEVA NB. 1949. Two species of methane-oxidizing mycobacteria. Mikrobiologiya 18:310–317.

[13] van Spanning RJM, Guan Q, Melkonian C, Gallant J, Polerecky L, Flot J-F, Brandt BW, Braster M, Iturbe Espinoza P, Aerts JW, Meima-Franke MM, Piersma SR, Bunduc CM, Ummels R, Pain A, Fleming EJ, van der Wel NN, Gherman VD, Sarbu SM, Bodelier PLE, Bitter W. 2022. Methanotrophy by a Mycobacterium species that dominates a cave microbial ecosystem. Nat Microbiol 7:2089–2100. doi:10.1038/s41564-022-01252-3 36329197

[14] Xu X, Xia Z, Liu Y, Liu E, Müller K, Wang H, Luo J, Wu X, Beiyuan J, Fang Z, Xu J, Di H, Li Y. 2021. Interactions between methanotrophs and ammonia oxidizers modulate the response of in situ methane emissions to simulated climate change and its legacy in an acidic soil. Science of The Total Environment 752:142225. doi:10.1016/j.scitotenv.2020.142225 33207503

[15] Yang Y, Tong T, Chen J, Liu Y, Xie S. 2020. Ammonium impacts methane oxidation and methanotrophic community in freshwater sediment. Front Bioeng Biotechnol 8:250. doi:10.3389/fbioe.2020.00250 32296693 PMC7137091

[16] Kambara H, Shinno T, Matsuura N, Matsushita S, Aoi Y, Kindaichi T, Ozaki N, Ohashi A. 2022. Environmental factors affecting the community of methane-oxidizing bacteria. Microbes Environ 37:ME21074. doi:10.1264/jsme2.ME21074 35342121 PMC8958294

[17] Martin M. 2011. Cutadapt removes adapter sequences from high-throughput sequencing reads. EMBnet j 17:10. doi:10.14806/ej.17.1.200

[18] Bolger AM, Lohse M, Usadel B. 2014. Trimmomatic: a flexible trimmer for Illumina sequence data. Bioinformatics 30:2114–2120. doi:10.1093/bioinformatics/btu170 24695404 PMC4103590

[19] Aronesty E. 2013. Comparison of sequencing utility programs. TOBIOIJ 7:1–8. doi:10.2174/1875036201307010001

[20] Hall M, Beiko RG. 2018. 16S rRNA gene analysis with QIIME2. Methods Mol Biol 1849:113–129. doi:10.1007/978-1-4939-8728-3_8 30298251

[21] Bolyen E, Rideout JR, Dillon MR, Bokulich NA, Abnet CC, Al-Ghalith GA, Alexander H, Alm EJ, Arumugam M, Asnicar F, et al.. 2019. Reproducible, interactive, scalable and extensible microbiome data science using QIIME 2. Nat Biotechnol 37:852–857. doi:10.1038/s41587-019-0209-9 31341288 PMC7015180

[22] Fujii N, Kuroda K, Narihiro T, Aoi Y, Ozaki N, Ohashi A, Kindaichi T. 2022. Metabolic potential of the superphylum Patescibacteria reconstructed from activated sludge samples from a municipal wastewater treatment plant. Microbes Environ 37:ME22012. doi:10.1264/jsme2.ME22012 35768268 PMC9530719

[23] Quast C, Pruesse E, Yilmaz P, Gerken J, Schweer T, Yarza P, Peplies J, Glöckner FO. 2013. The SILVA ribosomal RNA gene database project: improved data processing and web-based tools. Nucleic Acids Res 41:D590–6. doi:10.1093/nar/gks1219 23193283 PMC3531112

[24] Epperson LE, Strong M. 2020. A scalable, efficient, and safe method to prepare high quality DNA from mycobacteria and other challenging cells. J Clin Tuberc Other Mycobact Dis 19:100150. doi:10.1016/j.jctube.2020.100150 32154387 PMC7052505

[25] Zhang J, Kobert K, Flouri T, Stamatakis A. 2014. PEAR: a fast and accurate Illumina Paired-End reAd mergeR. Bioinformatics 30:614–620. doi:10.1093/bioinformatics/btt593 24142950 PMC3933873

[26] Langmead B, Trapnell C, Pop M, Salzberg SL. 2009. Ultrafast and memory-efficient alignment of short DNA sequences to the human genome. Genome Biol 10:R25. doi:10.1186/gb-2009-10-3-r25 19261174 PMC2690996

[27] Kircher M, Sawyer S, Meyer M. 2012. Double indexing overcomes inaccuracies in multiplex sequencing on the Illumina platform. Nucleic Acids Res 40:e3–e3. doi:10.1093/nar/gkr771 22021376 PMC3245947

[28] Mitra A, Skrzypczak M, Ginalski K, Rowicka M. 2015. Strategies for achieving high sequencing accuracy for low diversity samples and avoiding sample bleeding using illumina platform. PLOS ONE 10:e0120520. doi:10.1371/journal.pone.0120520 25860802 PMC4393298

[29] Guenay-Greunke Y, Bohan DA, Traugott M, Wallinger C. 2021. Handling of targeted amplicon sequencing data focusing on index hopping and demultiplexing using a nested metabarcoding approach in ecology. Sci Rep 11:19510. doi:10.1038/s41598-021-98018-4 34593851 PMC8484467

[30] Sinha R, Stanley G, Gulati GS, Ezran C, Travaglini KJ, Wei E, Chan CKF, Nabhan AN, Su T, Morganti RM, Conley SD, Chaib H, Red-Horse K, Longaker MT, Snyder MP, Krasnow MA, Weissman IL. 2017. Index switching causes “spreading-of-signalamong multiplexed samples in Illumina HiSeq 4000 DNA sequencing. bioRxiv. doi:10.1101/125724:125724

[31] Miyazaki M, Sakai S, Ritalahti KM, Saito Y, Yamanaka Y, Saito Y, Tame A, Uematsu K, Löffler FE, Takai K, Imachi H. 2014. Sphaerochaeta multiformis sp. nov., an anaerobic, psychrophilic bacterium isolated from subseafloor sediment, and emended description of the genus Sphaerochaeta. Int J Syst Evol Microbiol 64:4147–4154. doi:10.1099/ijs.0.068148-0 25249566

[32] Dedysh SN, Dunfield PF. 2011. Chapter three - Facultative and obligate methanotrophs: how to identify and differentiate them, p 31–44. In Rosenzweig AC, Ragsdale SW (ed), Methods in enzymology. Academic Press. 10.1016/B978-0-12-386905-0.00003-6 21419913

[33] Ludwig W. 2004. ARB: a software environment for sequence data. Nucleic Acids Res 32:1363–1371. doi:10.1093/nar/gkh293 14985472 PMC390282

[34] Kolmogorov M, Yuan J, Lin Y, Pevzner PA. 2019. Assembly of long, error-prone reads using repeat graphs. Nat Biotechnol 37:540–546. doi:10.1038/s41587-019-0072-8 30936562

[35] Wick RR, Holt KE. 2022. Polypolish: Short-read polishing of long-read bacterial genome assemblies. PLOS Comput Biol 18:e1009802. doi:10.1371/journal.pcbi.1009802 35073327 PMC8812927

[36] Hyatt D, Chen G-L, Locascio PF, Land ML, Larimer FW, Hauser LJ. 2010. Prodigal: prokaryotic gene recognition and translation initiation site identification. BMC Bioinformatics 11:119. doi:10.1186/1471-2105-11-119 20211023 PMC2848648

[37] Buchfink B, Xie C, Huson DH. 2015. Fast and sensitive protein alignment using DIAMOND. Nat Methods 12:59–60. doi:10.1038/nmeth.3176 25402007

[38] Huerta-Cepas J, Szklarczyk D, Heller D, Hernández-Plaza A, Forslund SK, Cook H, Mende DR, Letunic I, Rattei T, Jensen Lars J, Mering C, Bork P. 2018. eggNOG 5.0: a hierarchical, functionally and phylogenetically annotated orthology resource based on 5090 organisms and 2502 viruses. Nucleic Acids Res. doi:10.1093/nar/gky1085 PMC6324079 30418610

[39] Cantalapiedra CP, Hernández-Plaza A, Letunic I, Bork P, Huerta-Cepas J. 2021. eggNOG-mapper v2: functional annotation, orthology assignments, and domain prediction at the metagenomic scale. Mol Biol Evol 38:5825–5829. doi:10.1093/molbev/msab293 34597405 PMC8662613

[40] Katoh K, Standley DM. 2013. MAFFT multiple sequence alignment software version 7: improvements in performance and usability. Mol Biol Evol 30:772–780. doi:10.1093/molbev/mst010 23329690 PMC3603318

[41] Minh BQ, Schmidt HA, Chernomor O, Schrempf D, Woodhams MD, von Haeseler A, Lanfear R. 2020. IQ-TREE 2: new models and efficient methods for phylogenetic inference in the genomic era. Mol Biol Evol 37:1530–1534. doi:10.1093/molbev/msaa015 32011700 PMC7182206

[42] Lemoine F, Domelevo Entfellner J-B, Wilkinson E, Correia D, Dávila Felipe M, De Oliveira T, Gascuel O. 2018. Renewing Felsenstein’s phylogenetic bootstrap in the era of big data. Nature 556:452–456. doi:10.1038/s41586-018-0043-0 29670290 PMC6030568

[43] Parks DH, Chuvochina M, Rinke C, Mussig AJ, Chaumeil P-A, Hugenholtz P. 2022. GTDB: an ongoing census of bacterial and archaeal diversity through a phylogenetically consistent, rank normalized and complete genome-based taxonomy. Nucleic Acids Res 50:D785–D794. doi:10.1093/nar/gkab776 34520557 PMC8728215

[44] Criscuolo A, Gribaldo S. 2010. BMGE (Block Mapping and Gathering with Entropy): a new software for selection of phylogenetic informative regions from multiple sequence alignments. BMC Evol Biol 10:210. doi:10.1186/1471-2148-10-210 20626897 PMC3017758

[45] Lagkouvardos I, Joseph D, Kapfhammer M, Giritli S, Horn M, Haller D, Clavel T. 2016. IMNGS: a comprehensive open resource of processed 16S rRNA microbial profiles for ecology and diversity studies. Sci Rep 6:33721. doi:10.1038/srep33721 27659943 PMC5034312

[46] Vorobev AV, Baani M, Doronina NV, Brady AL, Liesack W, Dunfield PF, Dedysh SN. 2011. Methyloferula stellata gen. nov., sp. nov., an acidophilic, obligately methanotrophic bacterium that possesses only a soluble methane monooxygenase. Int J Syst Evol Microbiol 61:2456–2463. doi:10.1099/ijs.0.028118-0 21097638

[47] Vekeman B, Kerckhof F-M, Cremers G, de Vos P, Vandamme P, Boon N, Op den Camp HJM, Heylen K. 2016. New methyloceanibacter diversity from North Sea sediments includes methanotroph containing solely the soluble methane monooxygenase. Environ Microbiol 18:4523–4536. doi:10.1111/1462-2920.13485 27501305

[48] Khadem AF, Pol A, Wieczorek A, Mohammadi SS, Francoijs K-J, Stunnenberg HG, Jetten MSM, Op den Camp HJM. 2011. Autotrophic methanotrophy in verrucomicrobia: Methylacidiphilum fumariolicum SolV uses the Calvin-Benson-Bassham cycle for carbon dioxide fixation. J Bacteriol 193:4438–4446. doi:10.1128/JB.00407-11 21725016 PMC3165502

[49] Rasigraf O, Kool DM, Jetten MSM, Sinninghe Damsté JS, Ettwig KF. 2014. Autotrophic carbon dioxide fixation via the Calvin-Benson-Bassham cycle by the denitrifying methanotroph “Candidatus Methylomirabilis oxyfera”. Appl Environ Microbiol 80:2451–2460. doi:10.1128/AEM.04199-13 24509918 PMC3993179

[50] Baxter NJ, Hirt RP, Bodrossy L, Kovacs KL, Embley TM, Prosser JI, Murrell JC. 2002. The ribulose-1,5-bisphosphate carboxylase/oxygenase gene cluster of Methylococcus capsulatus (Bath). Arch Microbiol 177:279–289. doi:10.1007/s00203-001-0387-x 11889481

[51] He R, Chen M, Ma RC, Su Y, Zhang X. 2017. Ammonium conversion and its feedback effect on methane oxidation of Methylosinus sporium. J Biosci Bioeng 123:466–473. doi:10.1016/j.jbiosc.2016.11.003 27939869

[52] Guo K, Hakobyan A, Glatter T, Paczia N, Liesack W. 2022. Methylocystis sp. strain SC2 acclimatizes to increasing NH4+ levels by a precise rebalancing of enzymes and osmolyte composition mSystems 7:e0040322. doi:10.1128/msystems.00403-22 PMC9600857 36154142

[53] Khadem AF, Pol A, Jetten MSM, Op den Camp HJM. 2010. Nitrogen fixation by the verrucomicrobial methanotroph “Methylacidiphilum fumariolicum” SolV. Microbiology (Reading) 156:1052–1059. doi:10.1099/mic.0.036061-0 20056702

[54] King GM, Schnell S. 1994. Ammonium and nitrite inhibition of methane oxidation by methylobacter albus BG8 and Methylosinus trichosporium OB3b at low methane concentrations. Appl Environ Microbiol 60:3508–3513. doi:10.1128/aem.60.10.3508-3513.1994 16349402 PMC201847

[55] Nyerges G, Stein LY. 2009. Ammonia cometabolism and product inhibition vary considerably among species of methanotrophic bacteria. FEMS Microbiol Lett 297:131–136. doi:10.1111/j.1574-6968.2009.01674.x 19566684

[56] King GM, Schnell S. 1998. Effects of ammonium and non-ammonium salt additions on methane oxidation by Methylosinus trichosporium OB3b and maine forest soils. Appl Environ Microbiol 64:253–257. doi:10.1128/AEM.64.1.253-257.1998 16349485 PMC124702

[57] Versantvoort W, Pol A, Jetten MSM, van Niftrik L, Reimann J, Kartal B, Op den Camp HJM. 2020. Multiheme hydroxylamine oxidoreductases produce NO during ammonia oxidation in methanotrophs. Proc Natl Acad Sci USA 117:24459–24463. doi:10.1073/pnas.2011299117 32913059 PMC7533708

[58] Schmitz RA, Peeters SH, Versantvoort W, Picone N, Pol A, Jetten MSM, Op den Camp HJM. 2021. Verrucomicrobial methanotrophs: ecophysiology of metabolically versatile acidophiles. FEMS Microbiol Rev 45:fuab007. doi:10.1093/femsre/fuab007 33524112 PMC8498564

[59] King GM, Schnell S. 1994. Effect of increasing atmospheric methane concentration on ammonium inhibition of soil methane consumption. Nature 370:282–284. doi:10.1038/370282a0

[60] Long Y-Y, Liao Y, Miao J-Y, Shen D-S. 2014. Effects of ammonia on methane oxidation in landfill cover materials. Environ Sci Pollut Res Int 21:911–920. doi:10.1007/s11356-013-1963-8 23832775

[61] Mosier A, Schimel D, Valentine D, Bronson K, Parton W. 1991. Methane and nitrous oxide fluxes in native, fertilized and cultivated grasslands. Nature 350:330–332. doi:10.1038/350330a0

[62] Nesbit SP, Breitenbeck GA. 1992. A laboratory study of factors influencing methane uptake by soils. Agriculture, Ecosystems & Environment 41:39–54. doi:10.1016/0167-8809(92)90178-E

[63] Martín MT, Valdepeñas Polo L, González Yélamos J, Cuevas Rodríguez J. 2023. Ammonium concentration in stream sediments resulting from decades of discharge from a wastewater treatment plant. Heliyon 9:e21860. doi:10.1016/j.heliyon.2023.e21860 38027734 PMC10660492

[64] WHO. 2021. A global overview of national regulations and standards for drinking-water quality

[65] Gouzy A, Healy C, Black KA, Rhee KY, Ehrt S. 2021. Growth of Mycobacterium tuberculosis at acidic pH depends on lipid assimilation and is accompanied by reduced GAPDH activity. Proc Natl Acad Sci USA 118. doi:10.1073/pnas.2024571118 PMC8364206 34341117

[66] Ling F, Hwang C, LeChevallier MW, Andersen GL, Liu W-T. 2016. Core-satellite populations and seasonality of water meter biofilms in a metropolitan drinking water distribution system. ISME J 10:582–595. doi:10.1038/ismej.2015.136 26251872 PMC4817684

[67] van der Wielen PWJJ, Voost S, van der Kooij D. 2009. Ammonia-oxidizing bacteria and archaea in groundwater treatment and drinking water distribution systems. Appl Environ Microbiol 75:4687–4695. doi:10.1128/AEM.00387-09 19465520 PMC2708422

