# Supplementary material for: First isolation of a methanotrophic Mycobacterium reveals ammonia- and pH-tolerant methane oxidation
Source: Appl Environ Microbiol. 2025 Jul 31;91(8):e00796-25. doi: 10.1128/aem.00796-25 (PMC12366331; doi:10.1128/aem.00796-25)
Supplement: Supplemental material, part I — Fig. S1 to S10, Tables S1 and S2, and Notes S1 and S2. [file aem.00796-25-s0001.docx]

**Supplementary information**

**First isolation of a methanotrophic *Mycobacterium* reveals ammonia- and pH-tolerant methane oxidation**

Hiromi Kambara^1,2,*^#, Taito Kawamoto^2^, Shuji Matsushita^3^, Tomonori Kindaichi^2^, Noriatsu Ozaki^2,*^, Yoshiteru Aoi^4^, Yoshihiro Takaki^1^, Hiroyuki Imachi^1^, Masaru Konishi Nobu^1^, Miyuki Ogawara^1^, Akiyoshi Ohashi^2^#

^1^Institute for Extra-cutting-edge Science and Technology Avant-garde Research (X-star), Japan Agency for Marine-Earth Science and Technology (JAMSTEC), Yokosuka, Kanagawa, Japan

^2^Department of Civil and Environmental Engineering, Graduate School of Advanced Science and Engineering, Hiroshima University, Higashihiroshima, Hiroshima, Japan

^3^Agricultural Technology Research Center, Hiroshima Prefectural Technology Research Institute, Higashihiroshima. Hiroshima, Japan

^4^Program of Biotechnology, Graduate School of Integrated Sciences for Life, Hiroshima University, Higashihiroshima, Hiroshima, Japan.

^*^Present address for Hiromi Kambara: Biomanufacturing Process Research Center, National Institute of Advanced Industrial Science and Technology (AIST), Sapporo, Hokkaido, Japan

^*^Present address for Noriatsu Ozaki: Life and Environmental Sciences, Prefectural University of Hiroshima, Shobara, Hiroshima, Japan

**# Corresponding authors:**

**Hiromi Kambara**, Email: [hiromi.kambara@aist.go.jp](mailto:hiromi.kambara@aist.go.jp)

**Akiyoshi Ohashi**, Email: [ecoakiyo@hiroshima-u.ac.jp](mailto:ecoakiyo@hiroshima-u.ac.jp)

**Content**

Supplementary Figures S1–S10

Supplementary Tables S1 and S2

Supplementary Notes S1 and S2

Supplementary References

**Supplementary Figures**


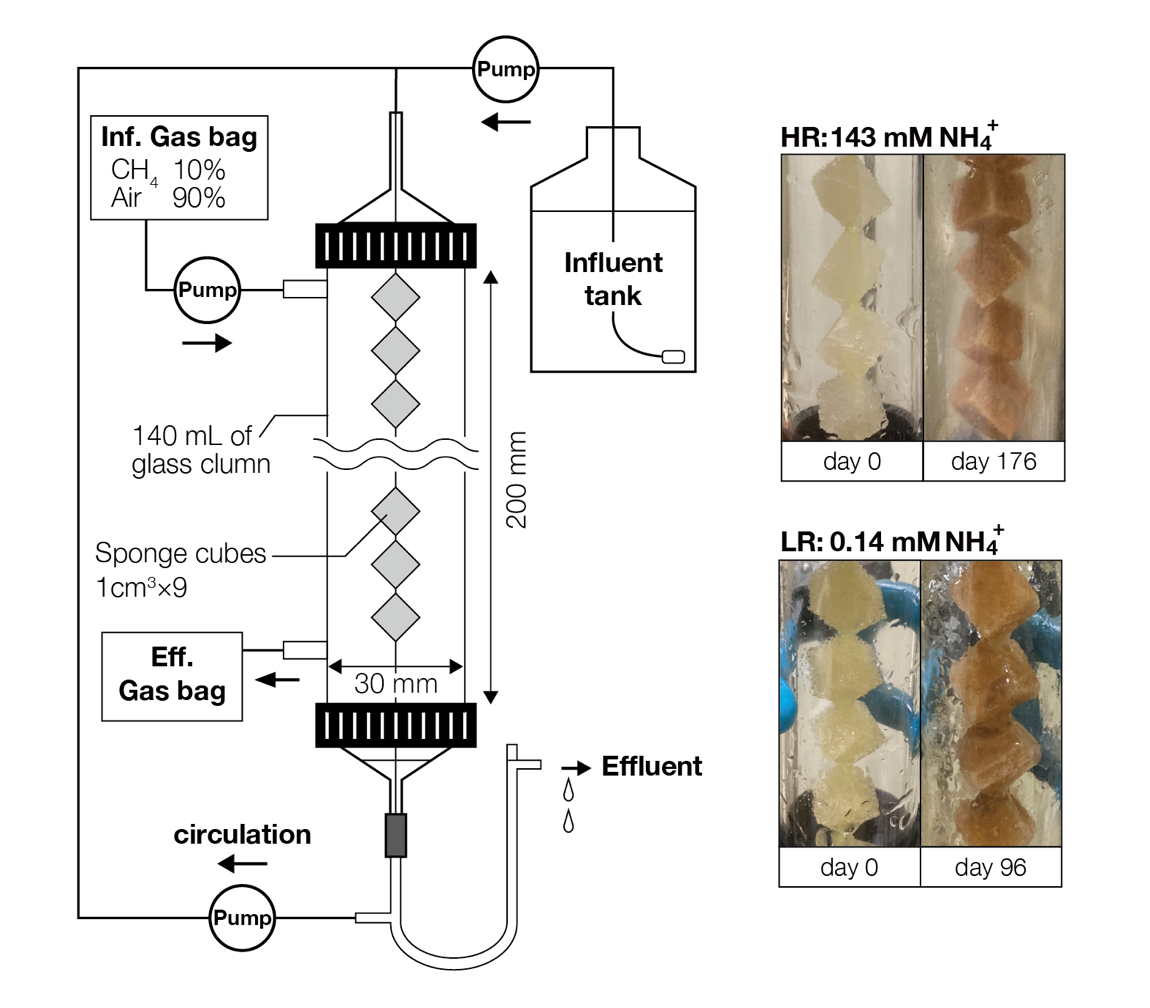


**Fig. S1** **Schematic diagram of the closed DHS reactor and photographs of sponge carriers from HR and LR.**


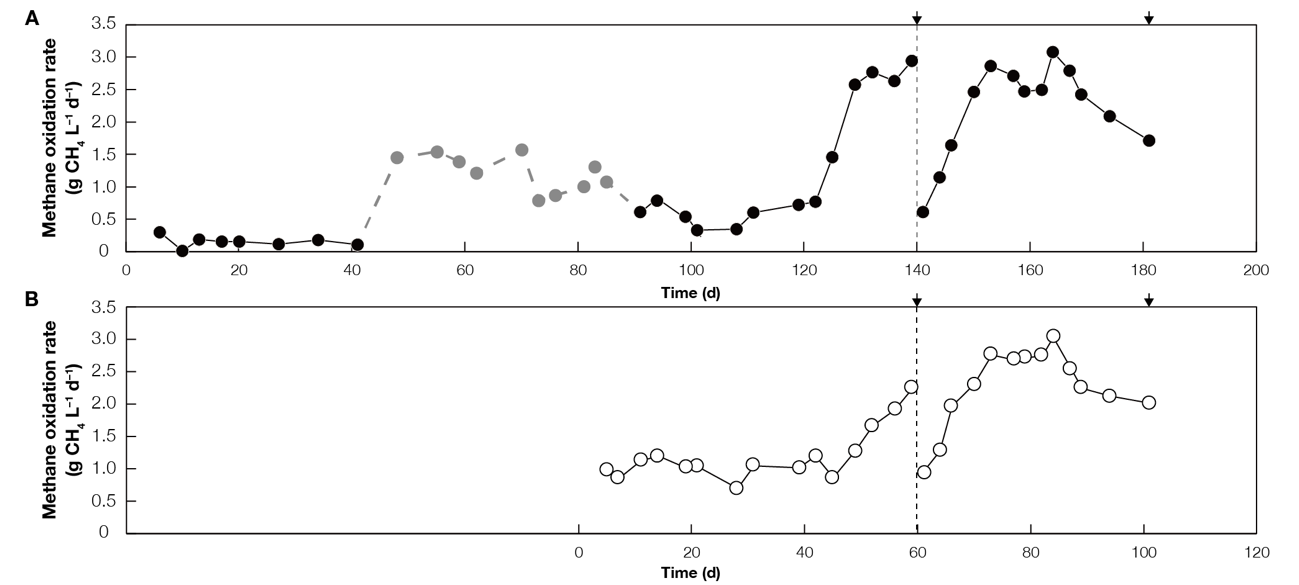


**Fig. S2** **Time courses of methane oxidation rates of (A) reactor HR (143 mM NH_4_^+^) and (B) reactor LR (0.14 mM NH_4_^+^).** The dotted line with grey plots in panel **A** represents the period of gas leakage, which resulted in an apparent elevation of methane oxidation rate. Arrows indicate the days when the biomass samples were collected. The dotted line represents the day of restarting the operation. The apparent methane oxidation rates of HR and LR exhibited a decrease from day 169 and 89, respectively, which was attributed to an increase in loading rate rather than an actual reduction in methane oxidation efficiency.


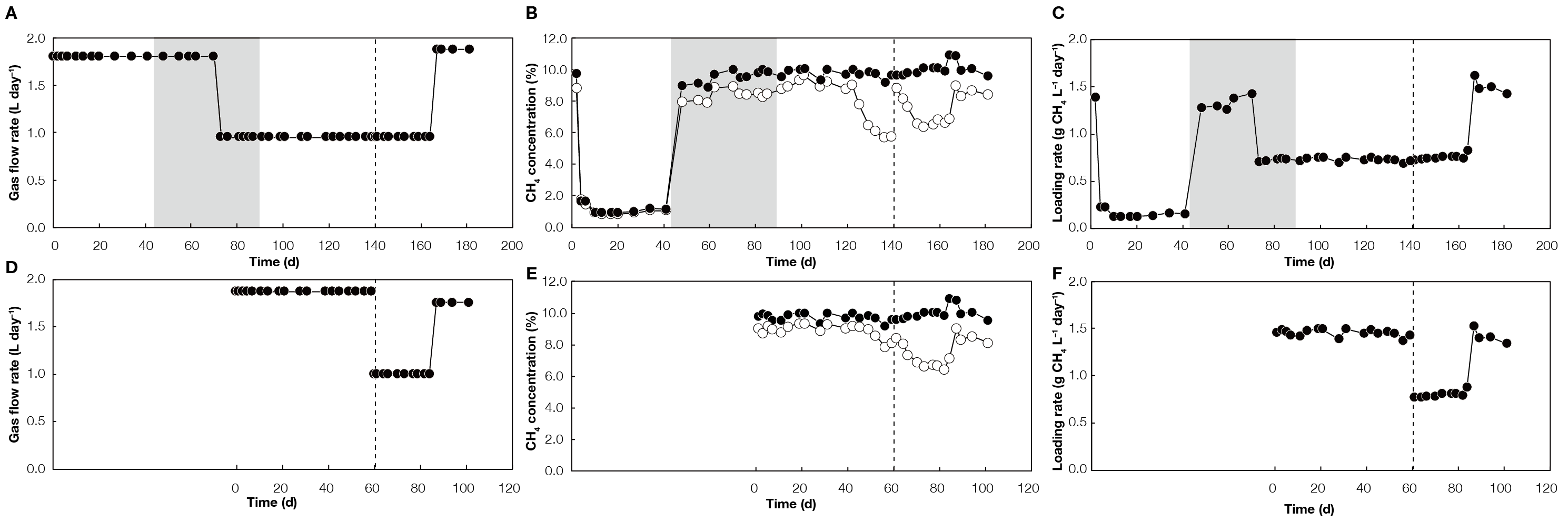


**Fig. S3** **Time course of gas flow rate, methane concentration of influent and effluent, and methane loading rate based on sponge volume of HR (A–C) and LR (D–F).**

A filled circle and open circle in **B** and **E** indicate methane concentrations of influent and effluent, respectively. The dotted line represents the day of restarting operations. The gray areas in panels **A**–**C** correspond to the period of gas leakage representing a dotted line with the gray plot in Fig. S2A. Loading rates were calculated using the following equations.

$$Loadingrate\left( g{CH}_{4}L^{-1}{day}^{-1} \right)=\frac{{{CH}_{4}\left( \text{\%} \right)}_{Inf.}}{100}\times\frac{Q}{V_{sponge}}\times\frac{M_{{CH}_{4}}}{V_{m}}$$

V_m_ (L mol^−1^): Molecular volume

M_CH4_ (g mol^−1^): Molar mass of methane

V_sponge_ (L): Total sponge volume

Q: Flow rate (L day^−1^)


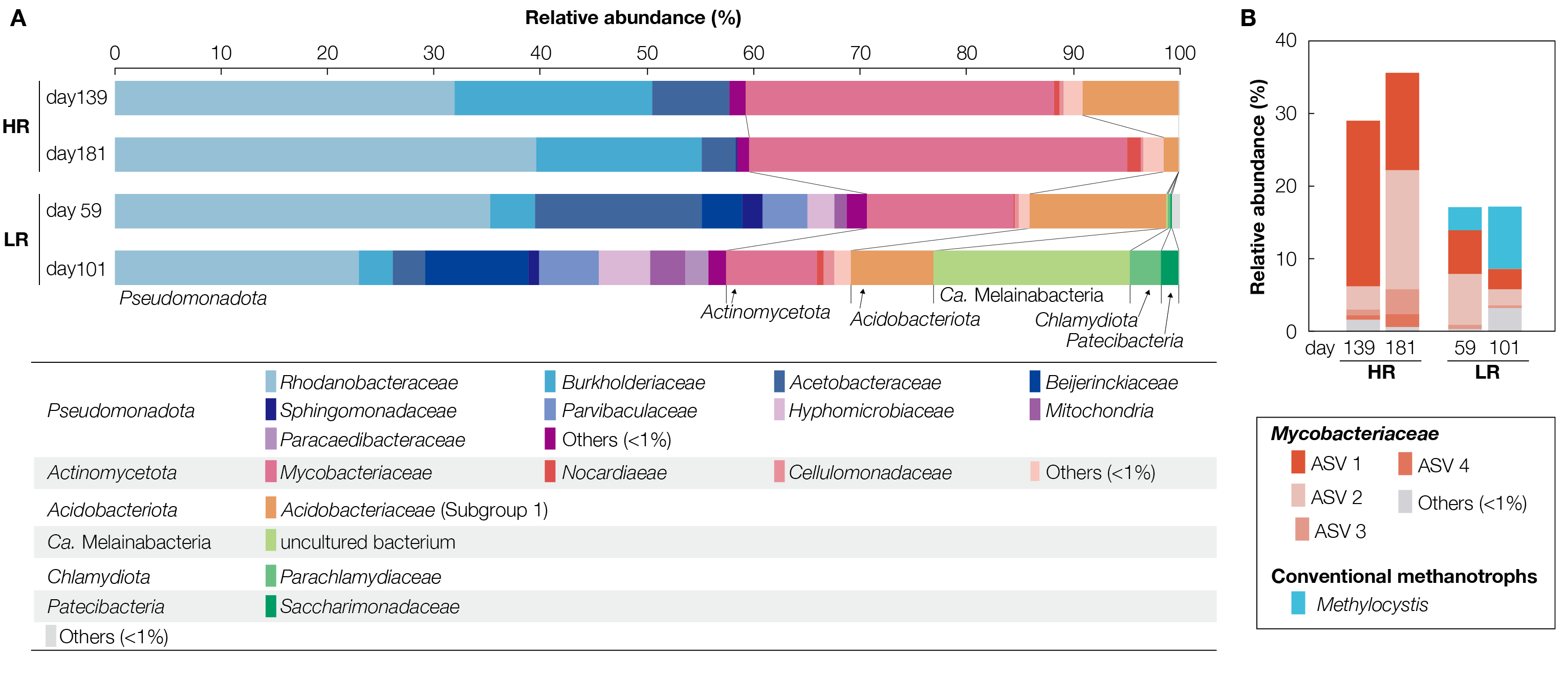


**Fig. S4** **Microbial community compositions in reactors HR and LR based on 16S rRNA gene amplicon sequencing analysis**. **A,** Family-level taxonomic composition. **B,** Relative abundance of ASVs belonging to *Mycobacteriaceae* and conventional methanotrophs.

**Fig. S5 MM-1 colonies on plate with different concentrations of NH_4_^+^.** **A,** 14.3 mM. **B,** 143 mM.


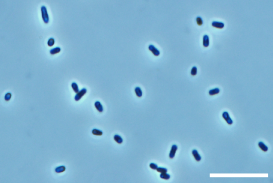


**Fig. S6 Photomicrograph of MM–1 grown on methane at 7.1 mM.** The bar represents 10 μm.


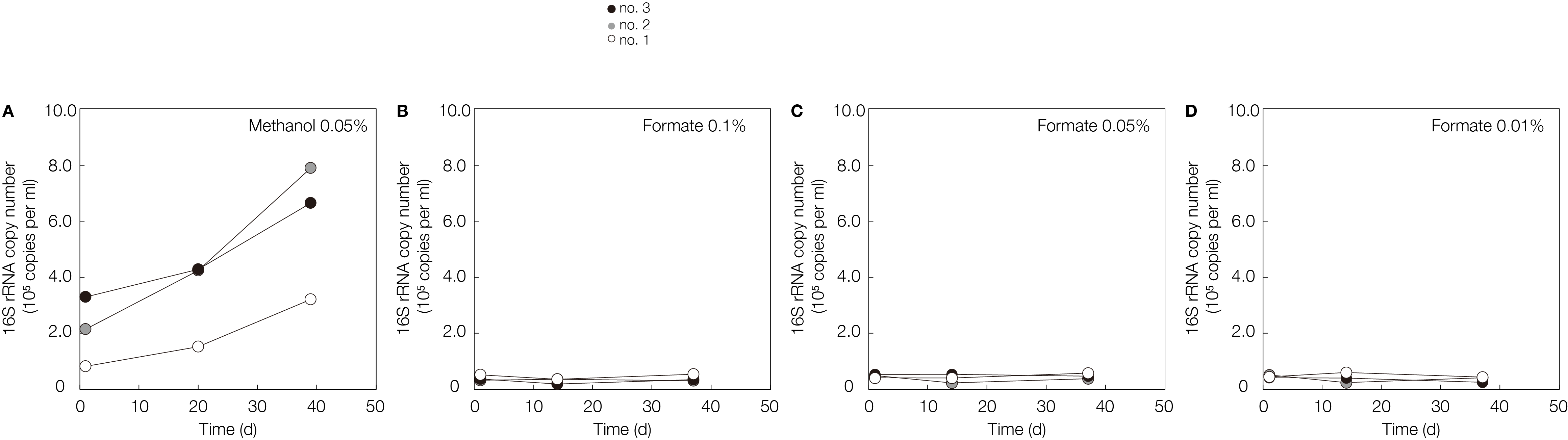


**Fig. S7 Growth curves of MM-1 with methanol (A) or formate (B–D) as the provided substrate.**


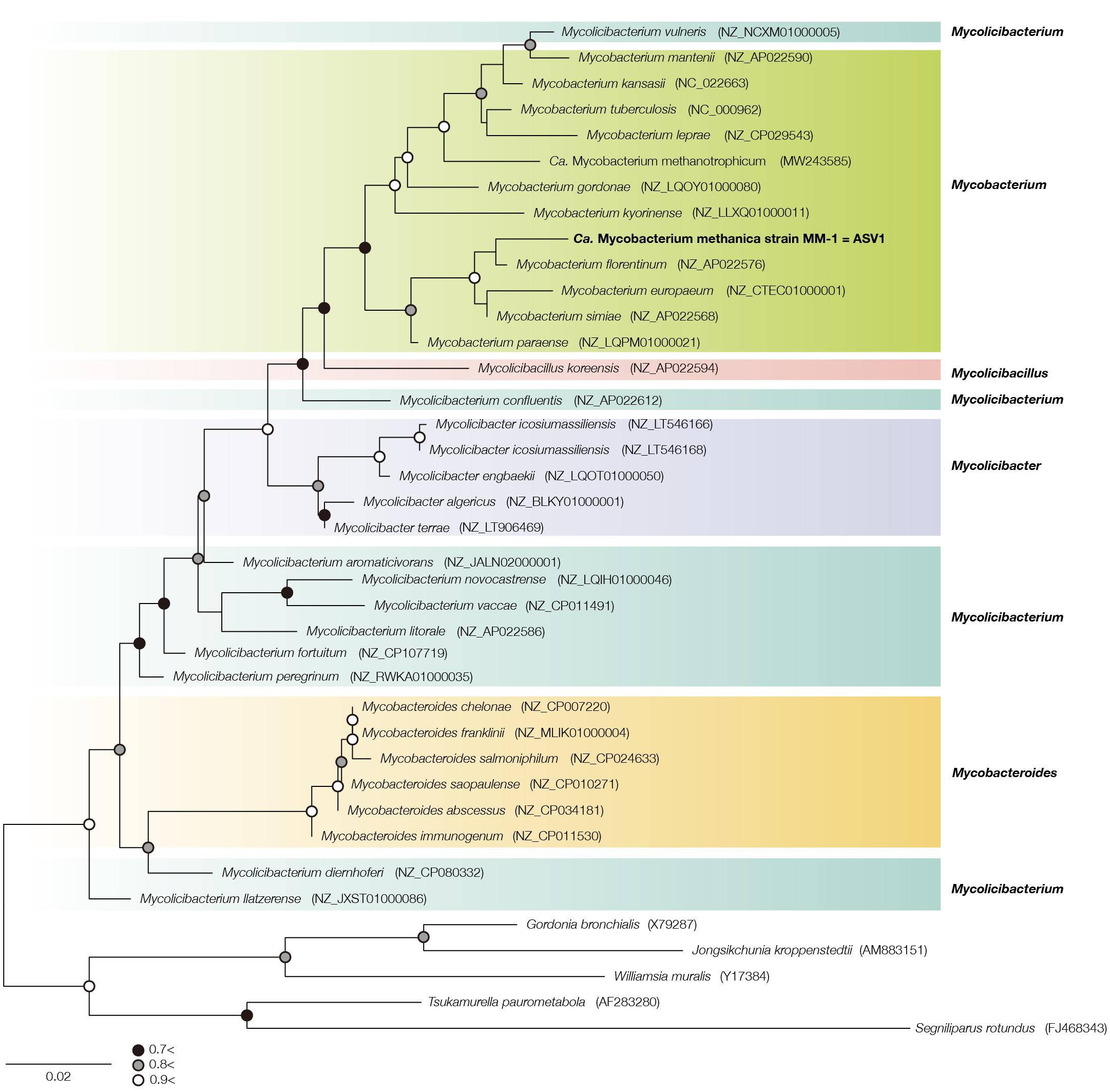


**Fig. S8** **Phylogenetic tree based on 16S rRNA genes.** The terms of parentheses represent the accession number.

**Fig. S9** **Unrooted phylogenetic tree based on *mmoX*.** The terms of parentheses represent the accession number.


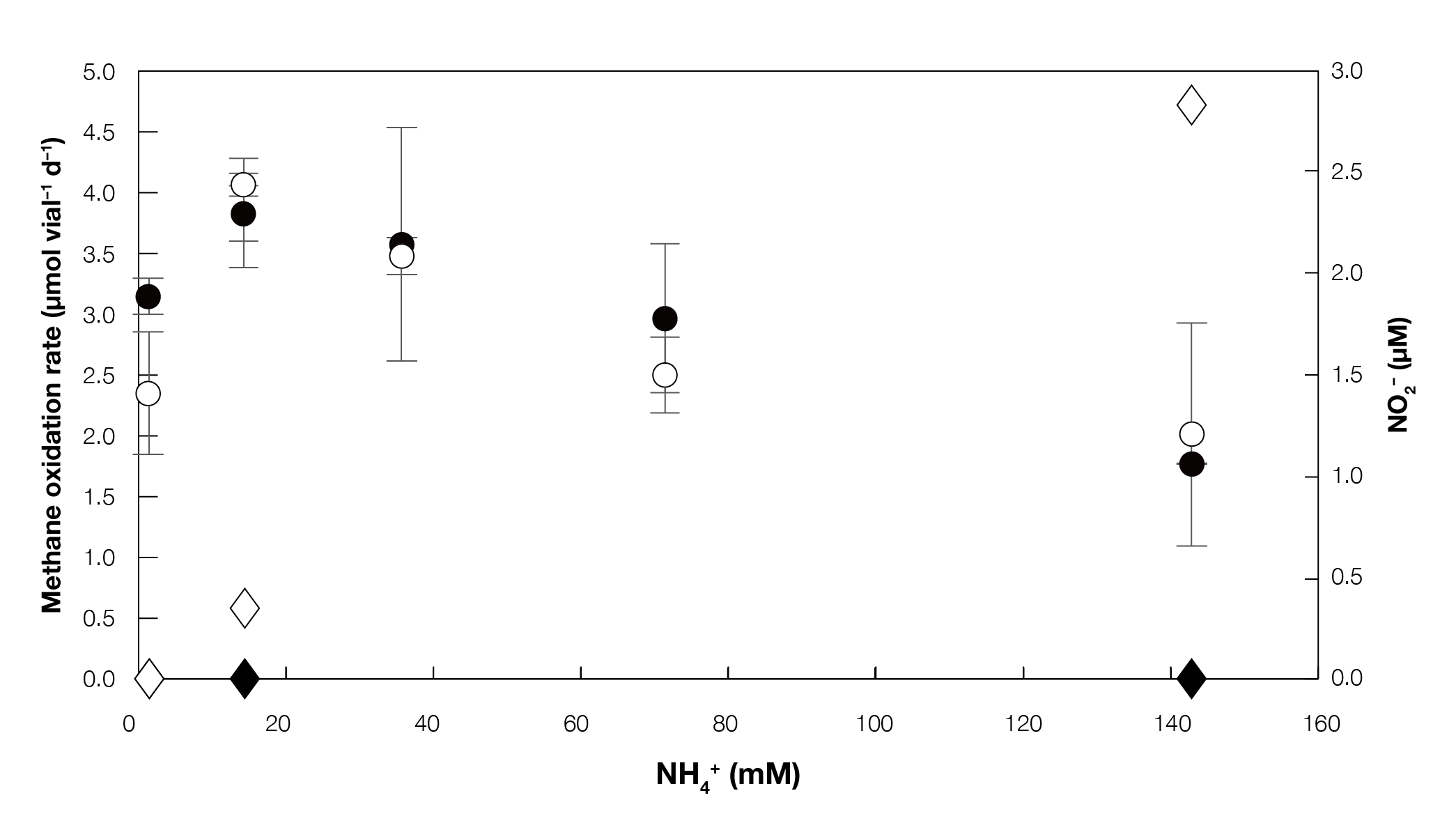


**Fig. S10** **Methane oxidation rate at pH 4 and 7, and NO_2_^–^ concentration at the end of methane oxidation test.** The filled and open circles represent the methane oxidation rate at pH 4 and 7, respectively (n=3). The filled and open diamonds indicate NO_2_^–^ concentration at pH 4 and 7, respectively.

**Supplementary Table**

**Table S1** **Composition of trace element solution (grams per liter).**

| Mineral solution | |
| --- | --- |
| CaCl_2_·2H_2_O | 5 |
| MgCl_2_·6H_2_O | 33 |
| KCl | 16 |
| Trace elements solution | |
| CoCl_2_·6H_2_O | 0.17 |
| ZnSO_4_·7H_2_O | 0.15 |
| H_3_BO_3_ | 0.06 |
| MnCl·4H_2_O | 0.04 |
| CuCl_2_·2H_2_O | 0.027 |
| Na_2_MoO_4_·2H_2_O | 0.025 |
| AlCl_3_ | 0.013 |
| NiCl_2_·6H_2_O | 0.024 |
| Na_2_SeO_4_ | 0.0017 |
| Na_2_WO_4_・2H_2_O | 0.0033 |
| FeSO_4_・7H_2_O solution | |
| FeSO_4_・7H_2_O | 5.49 |
| KNO_3_ solution | |
| KNO_3_ | 7.25 |
| KH_2_PO_4_ solution | |
| KH_2_PO_4_ | 8.29 |

**Table S2 Primer sets used for amplicon sequencing to evaluate the purity of the isolated strain MM-1 (1)**

| Name | Sequence (5'-3') |
| --- | --- |
| 530F mixture |  |
| Bac 530F | GTGCCAGCAGCCGCGG |
| Arch 530F | GTGBCAGCCGCCGCGG |
| Arch2 530F | YTGCCAGCCGCCGCGG |
| Bac2 530F | GTGCCAGCAGCWGCGG |
| Bac3 530F | GTGCCAGCAGTCGCGG |
| Bac4 530F | GTGCCAGAAGMMTCGG |
| Nano 530F | GTGGCAGTCGCCACGG |
| 907R mixture |  |
| Uni 907R | CCGYCAATTCMTTTRAGTTT |
| DeepAB 907R | CCGYCTATTCCTTTGAGTTT |
| SAG-Del 907R | CCGYCAATTTCTTTRAGTTT |
| DeepAB2 907R | CCGYCAATTCCCTTRAGTTT |
| Arch2 907R | CCGYCAATTCCTTMAAGTTT |
| OP11 907R | CCGCCAATTCCTTTGAATTT |

**Supplementary Notes**

**Supplementary Note S1**

**Bioreactor operation and microbial community shifts.**

Two DHS reactors, HR and LR, inoculated with activated sludge were operated at high (143 mM) and low (0.14 mM) NH_4_^+^ concentrations, respectively. HR operation was started 3 months before LR operation (Fig. S2) as our previous study revealed that methane oxidation begins earlier at lower NH_4_^+^ concentrations than at higher concentrations (2). In HR, no clear methane oxidation was noted over approximately 120 days (Fig. S2A). An increase in methane oxidation rate from days 48 to 90 was attributed to gas leakage from the reactor. However, a distinct increase in methane oxidation was observed after day 125. On day 125, there was a significant decrease in methane concentration in HR from 9.7% to 7.8% (Fig. S3B), which roughly corresponded to the methane oxidation rate based on the sponge volume (R_m_). = 1.5 g CH_4_ L^−1^ d^−1^ (Fig. S2A). Methane concentrations in the effluent decreased continually, and R_m_ then reached a plateau of 2.6 g CH_4_ L^−1^ d^−1^ on day 129. Conversely, LR displayed methane oxidation from day 52 (Fig. S2B), reaching 2.2 g CH_4_ L^−1^ d^−1^ on day 59, which was almost similar to that noted in HR on day 129.

To enhance the enrichment of methanotrophs, reactor operations were paused, biomass was collected from the sponge carriers, 250-fold diluted biomass was transferred to new sponge carriers, and then both reactors were restarted. Methane oxidation was observed in both reactors after a shorter operational period than in the previous reactor operations (Fig. S2), indicating that methanotrophs were further enriched in both reactors. To further methanotroph enrichment, the gas flow rate was increased from 1.0 to 1.8 L^−1^ d^−1^ (Fig. S3A and S3D). However, the methane oxidation rate did not increase in both reactors (Fig. 2S). Therefore, the reactors were shut down and biomass samples were collected to analyze the microbial community and isolate methanotrophs.

For reactor HR, 16S rRNA gene sequence analysis yielded 41 and 44 amplicon sequence variants (ASVs) from 22,569 and 17,501 sequence reads for biomass samples on days 139 and 181, respectively. The numbers of ASVs with a population size of >1% were 12 and 13, respectively. Dominant ASVs were primarily associated with three phyla: *Acidobacteriota* (day 139, 9.0%; day 181, 1.4%), *Actinomycetota* (day 139, 31.7%; day 181, 38.9%), and *Pseudomonadota* (day 139, 59.3%; day 181, 59.5%), indicating similar microbial community structures at the phylum level across both time points (Fig. S4A). As for methanotrophs in HR, microbial community analysis revealed an absence of conventional methanotrophs belonging to *Pseudomonadota*, *Verrucomicrobiota*, and *Ca.* Methylomirabilota. Notably, *Mycobacteriaceae* was present in significant populations, constituting 29.0% of the population on day 139 and 35.6% on day 181 (Fig. S4B), hinting at novel methanotrophic species within this genus. Within the family *Mycobacteriaceae*, four dominant ASVs (>1%) (numbered 1 to 4) were observed, with ASVs 1 and 2 being particularly predominantly (Fig. S4B).

For reactor LR, 16S rRNA gene sequencing analysis resulted in the identification of 61 and 67 ASVs from 29,429 and 22,785 sequence reads for biomass samples on days 59 and 101, respectively. The numbers of dominant ASVs (>1%) were 17 and 23 for day 59 and 101 samples, respectively. These ASVs were primarily affiliated with six phyla: *Acidobacteriota* (day 59, 12.9%; day 101, 7.7%), *Actinomycetota* (day 59, 15.3%; day 101, 11.7%), *Chlamydiota* (day 59, 0.3%; day 101, 2.9%), *Ca*. Melainabacteria (day 59, 0.1%; day 101, 18.5%), *Patescibacteria* (day 59, 0.05%; day 101, 1.6%), and *Pseudomonadota* (day 59, 70.6%; day 101, 57.4%) (Fig. S4A). Microbial communities in LR were changed in relative abundance, with increases in *Chlamydiota*, *Ca*. Melainabacteria, and *Patescibacteria* following restarting the reactor. As for methanotrophs, *Methylocystis*, a conventional methanotroph, was the primary organism in LR biomass, with population sizes of 3.2% on day 59 and 9.1% on day 101 (Fig. S4B), indicating that methane was primarily consumed by conventional methanotrohps. *Mycobacteriaceae* was also detected well in the LR reactor, but its relative abundance was considerably lower (day 59, 13.8%; day 101, 8.5%) than that in HR.

Through these reactor operations, we confirmed the methane oxidation under high NH_4_^+^ conditions and revealed the differences in microbial community between high and low NH_4_^+^ environments. The collective evidence indicates that the presence of NH_4_^+^ influences the enrichment process of methanotrophs.

**Supplementary Note S2**

**Description of *Ca*. Mycobacterium methanica.**

**Etymology**. The species’ name,methanica; me.tha.ni.ca. N.L. neut. n. *methanum*, methane; L. fem. adj. suff. *-ica*, suffix used with the sense of pertaining to; N.L. fem. adj. *methanica*, related to or associated with methane.

**Locality**. Isolated from activated sludge obtained from an aeration tank at a municipal wastewater treatment plant in Higashi-Hiroshima, Japan.

**Diagnosis**. Aerobic, methane-oxidizing bacterium. Cells are rod, around 1.3 to 3.3 µm long and 1.0 µm wide. Its methane oxidation activity is maintained under up to 500 mM NH_4_^+^ at pH 4, and 143 mM at pH 7. It shows methane oxidation activity in a temperature range of 10 to 37 ºC and in a pH range of 0.75 to 8.0 at 14.3 mM NH_4_^+^.

**References**

1. Nunoura T, Takaki Y, Kazama H, Hirai M, Ashi J, Imachi H, Takai K. 2012. Microbial Diversity in Deep-sea Methane Seep Sediments Presented by SSU rRNA Gene Tag Sequencing. Microbes and Environments 27:382-390.

2. Epperson LE, Strong M. 2020. A scalable, efficient, and safe method to prepare high quality DNA from mycobacteria and other challenging cells. Journal of Clinical Tuberculosis and Other Mycobacterial Diseases 19:100150.
